# QTc Shortening After Oral Calcium Supplementation in a Poststroke Rehabilitation Patient Being Considered for Donepezil: A Case Report

**DOI:** 10.1002/ccr3.73206

**Published:** 2026-07-22

**Authors:** Benjamin Sims, Meghan Oram, Joshua Startup

**Affiliations:** ^1^ University of Michigan Ann Arbor Michigan USA

**Keywords:** donepezil, hypocalcemia, QTc interval, stroke rehabilitation

## Abstract

In a 70‐year‐old inpatient rehabilitation patient after multifocal ischemic stroke, QTc prolongation delayed consideration of donepezil for aphasia. Mild hypocalcemia was treated with oral calcium carbonate, after which QTc shortened from 464 to 412 ms. Calcium supplementation may support QTc optimization, though causality remains uncertain.

## Key Clinical Message

Oral calcium supplementation may represent a low‐risk adjunctive strategy for QTc optimization in selected poststroke rehabilitation patients with mild hypocalcemia who are being considered for QT‐prolonging medications, though causality cannot be inferred.

## 1 Introduction

Drug‐induced QTc prolongation is a key safety concern, and thresholds commonly used in practice classify QTc ≥ 450 ms in men and ≥ 460 ms in women as prolonged, with ≥ 500 ms conveying higher arrhythmic risk [1, 2, 3]. Accurate measurement and rate correction (e.g., Bazett, Fridericia) are essential [4]. Hypocalcemia prolongs the QTc, primarily through ST‐segment lengthening, and calcium replacement can shorten the interval [5, 6, 7]. Donepezil is associated with QTc prolongation and rare arrhythmia in post‐marketing reports and case series [3, 8, 9]; risk appears higher in the presence of other arrhythmia risk factors. In poststroke aphasia, small randomized trials suggest donepezil may improve language outcomes, but no pharmacotherapy is FDA‐approved for aphasia [3, 9].

## 2 Case History/Examination

A 70‐year‐old male was admitted to inpatient rehabilitation after an acute ischemic stroke with bilateral multifocal infarcts and a history of bilateral lung transplant on tacrolimus. Imaging revealed lesions in the bilateral centrum semiovale, left frontoparietal cortex, left temporal cortex, left putamen, and left cerebellum. His initial deficits included incomplete right hemiplegia, aphasia, dysarthria, impaired balance/gait, and cognitive/personality changes. There were no prior ECGs available for review prior to inpatient rehabilitation admission.

## 3 Differential Diagnosis, Investigations, and Treatment

**FIGURE 1:**
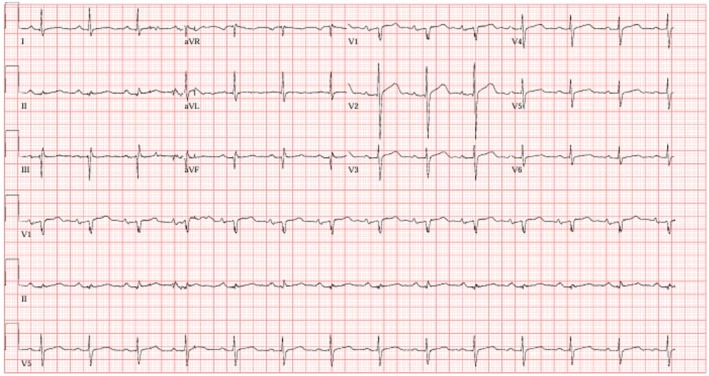
ECG on admission to inpatient rehabilitation.

**FIGURE 2:**
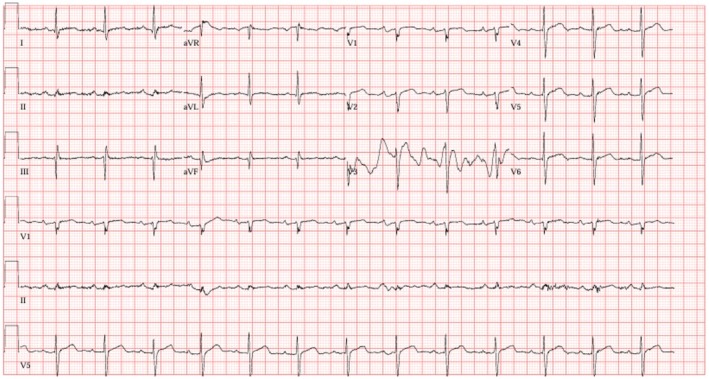
ECG on Day 6 of inpatient rehabilitation stay.

Routine ECG obtained on Day 0 of the rehabilitation stay, within the first hour of admission demonstrated normal sinus rhythm and a ventricular rate of 81 bpm with a QTc of 464 by machine report and consistent with Bazett's formula (Figure 1) [10]. During the initial evaluation with evident aphasia, donepezil was considered but ultimately deferred due to the prolonged QTc and the known potential of further QTc prolongation [6, 7, 8, 9]. Potentially reversible contributors of QTc prolongation were reviewed and on rehabilitation day 1 and laboratory analysis revealed a total calcium of 8.5 mg/dL with an albumin of 3.5 g/dL, corresponding to a corrected calcium of 8.9 mg/dL using the Payne formula (corrected calcium = measured calcium + 0.8 × (4.0 − albumin)) [11]. The institutional laboratory reference for total calcium was 8.6–10.3 mg/dL. Ionized calcium was not measured. On rehabilitation day 3, oral calcium carbonate 1250 mg, equivalent to 500 mg of elemental calcium per dose, twice daily was started in the evening to maximize correction of all electrolyte abnormalities. Six doses were received and on rehabilitation day 6, repeat ECG was performed approximately 1 h following dose 6 of calcium carbonate and demonstrated normal sinus rhythm with a ventricular rate of 80 bpm and a machine report and Bazett's confirmed QTc of 412 (Figure 2) [10]. No intermediate ECGs were available between the admission ECG and repeat ECG after calcium supplementation.

Other electrolyte factors were accounted for including a stable magnesium of 1.5 mg/dL throughout the stay, continuing oral magnesium supplementation with magnesium oxide 400 mg daily. Potassium ranged from 3.6 to 4.0 mmol/L following calcium initiation.

On rehabilitation day 6, the day of the repeat ECG, total calcium was 8.5 with an albumin of 3.3 g/dL, corresponding to a corrected calcium increased to 9.1 mg/dL using Payne's formula [11]. Medication review was notable for continued citalopram 40 mg daily, a QT‐relevant medication, without dose change during the interval between ECGs.

Ultimately, donepezil was not initiated due to the patient's aphasia clinically improving during the rehabilitation course.

## 4 Conclusion and Results

In this post‐stroke rehabilitation patient with mild hypocalcemia and prolonged QTc, oral calcium supplementation was followed by QTc shortening on repeat ECG. Because the total calcium level remained stable and the corrected calcium change was small, only two ECGs were used. Magnesium was low, but stable, and although no other medications were changed, other physiologic factors may have contributed, and direct cause cannot be established. Oral calcium supplementation may be considered as a broader component of QTc optimization in select patients where hypocalcemia is present and are being considered for QT‐prolonging medications, but further studies are necessary. Therefore, this case should be interpreted as an example of QTc optimization before possible initiation of QT‐prolonging therapy, rather than evidence that calcium supplementation enabled safe initiation of donepezil.

## 5 Discussion

Hypocalcemia prolongs QTc primarily via ST‐Segment lengthening and calcium replacement shortens QTc by restoring normal L‐type calcium channel inactivation kinetics [5, 6, 7]. Donepezil has documented associations with QTc prolongation and occasional arrhythmia, particularly when additional risk factors are present [3, 8, 9]. This case represented a patient who was admitted to inpatient rehabilitation with significant aphasia, prolonged QTc, and had multiple risk factors for QT prolongation including multiple electrolyte abnormalities on tacrolimus and a QT‐prolonging selective serotonin reuptake inhibitor [12]. With the severity of aphasia the patient experienced, he was considered for off‐label initiation of donepezil, but he ultimately clinically improved prior to administration. This case represents an opportunity to ask further controlled questions regarding electrolyte optimization in the setting of QT prolongation, in a mechanistically plausible fashion.

However, several limitations should limit the interpretation of this case. Although QTc decreased from 464 to 412 ms after oral calcium supplementation, the change in corrected calcium was small, and total calcium was unchanged. Therefore, the observed QTc improvement cannot be attributed definitively to calcium supplementation. Other factors may have contributed, including autonomic changes during stroke recovery, correction formula effects, intraindividual QT measurement variability, medication effect, electrolyte effects, or regression to the mean. Due to the short rehabilitation course, only two ECGs were measured, limiting assessment of ongoing temporal changes of the QTc. Finally, donepezil was not ultimately initiated due to clinical improvements; therefore, it does not demonstrate that calcium supplementation enabled safe initiation of a QT‐prolonging agent. The case rather highlights a potential approach to identifying and correcting a potentially reversible risk factor before considering QT‐prolonging therapy through a low‐risk intervention.

## Author Contributions

**Benjamin Sims:** writing – original draft, writing – review and editing, methodology, conceptualization. **Meghan Oram:** writing – original draft, conceptualization, writing – review and editing. **Joshua Startup:** supervision, writing – review and editing, project administration.

## Funding

The authors have nothing to report.

## Disclosure

Setting: Inpatient Rehabilitation Hospital.

## Consent

Written informed consent from the patient was obtained according to journal guidelines.

## Conflicts of Interest

The authors declare no conflicts of interest.

## Data Availability

The data that support the findings of this study are available on request from the corresponding author. The data are not publicly available due to privacy or ethical restrictions.
